# Algal extracellular release in river-floodplain dissolved organic matter: response of extracellular enzymatic activity during a post-flood period

**DOI:** 10.3389/fmicb.2015.00080

**Published:** 2015-02-17

**Authors:** Anna Sieczko, Maria Maschek, Peter Peduzzi

**Affiliations:** Department of Limnology and Bio-Oceanography, Inland Water Microbial and Viral Ecology, University of ViennaVienna, Austria

**Keywords:** extracellular enzymatic activity, photosynthetic extracellular release, autochthonous DOM, non-chromophoric DOM, river-floodplain

## Abstract

River-floodplain systems are susceptible to rapid hydrological events. Changing hydrological connectivity of the floodplain generates a broad range of conditions, from lentic to lotic. This creates a mixture of allochthonously and autochthonously derived dissolved organic matter (DOM). Autochthonous DOM, including photosynthetic extracellular release (PER), is an important source supporting bacterial secondary production (BSP). Nonetheless, no details are available regarding microbial extracellular enzymatic activity (EEA) as a response to PER under variable hydrological settings in river-floodplain systems. To investigate the relationship between bacterial and phytoplankton components, we therefore used EEA as a tool to track the microbial response to non-chromophoric, but reactive and ecologically important DOM. The study was conducted in three floodplain subsystems with distinct hydrological regimes (Danube Floodplain National Park, Austria). The focus was on the post-flood period. Enhanced %PER (up to 48% of primary production) in a hydrologically isolated subsystem was strongly correlated with β-glucosidase, which was related to BSP. This shows that—in disconnected floodplain backwaters with high terrestrial input—BSP can also be driven by autochthonous carbon sources (PER). In a semi-isolated section, in the presence of fresh labile material from primary producers, enhanced activity of phenol oxidase was observed. In frequently flooded river-floodplain systems, BSP was mainly driven by enzymatic degradation of particulate primary production. Our research demonstrates that EEA measurements are an excellent tool to describe the coupling between bacteria and phytoplankton, which cannot be deciphered when focusing solely on chromophoric DOM.

## Introduction

In river-floodplain systems seasonal floods or low water episodes create a range of lotic to lentic conditions in the backwaters. Depending on the duration and frequency of spates and on the distance of a side arm from the main channel, different types of side backwaters can be distinguished. These range from frequently connected to fragmented and very rarely flooded parts to side-arms that are completely disconnected from the main channel. Rapid hydrological events introduce substantial loads of terrestrial dissolved organic matter (DOM) into the floodplain (Hein et al., 2003). Moreover, disconnected and stable hydrological situations, observed after a flood, enhance phytoplankton productivity (Hein et al., 1999). This creates a complex DOM pool with a mixture of allochthonously and autochthonously derived material, potentially available for bacterial utilization.

Autochthonous autotrophic production is an important carbon source also for secondary production in rivers (Thorp and Delong, 2002; Bunn et al., 2003). Nonetheless, DOM generated *in situ* often is insufficient to exclusively support aquatic food webs (Kritzberg et al., 2004). Particularly in aquatic systems where primary production is low compared with the terrestrial load of DOC (Kritzberg et al., 2005), allochthonous DOC can become essential for bacterial secondary production (BSP) (Jansson et al., 2000). BSP may be much higher when faced with pulse resource addition compared to the same quantity of DOC supplied continuously over a longer time period (Lennon and Cottingham, 2008). This points to the importance of seasonal disturbance events (such as floods) for BSP.

Even if most of carbon needed for bacterial growth may be supplied from other than autochthonous sources (Fouilland and Mostajir, 2010), the autochthonous material is preferentially utilized prior to terrestrial DOM. In pelagic zones, an important source of autochthonous carbon is photosynthetic extracellular release of phytoplankton (PER). The link between PER and bacterial heterotrophic metabolism has been studied in freshwater and marine systems. In floodplain studies, however, this is rarely measured routinely. The rate and quality of the exudated material depends on the phytoplankton species, cell size (López-Sandoval et al., 2013) and on the type of aquatic ecosystem (Fogg, 1983). Nutrients (Meon and Kirchman, 2001; Wyatt et al., 2014), light (Hulatt et al., 2009), UV radiation (Pausz and Herndl, 1999) and other factors can also significantly impact the percentage, composition and microbial utilization of PER (Sarmento and Gasol, 2012; Landa et al., 2013). Related phytoplankton species may produce similar suits of DOM, which has implications for shaping bacterial communities (Becker et al., 2014). However, the composition of PER may depend on the growth phase of phytoplankton (Barofsky et al., 2009). In general, most primary production exudates are high-quality substrates, comprised mainly of monomeric sugars, carboxylic acids and amino acids (Bertilsson and Jones, 2003), which can be directly assimilated by bacteria, stimulating the growth and abundance of microbial communities (Norrman et al., 1995). Numerous studies have revealed the importance of PER for bacterial metabolism and the direct utilization of PER by bacteria (Cole, 1982; Chrzanowski and Hubbard, 1989; Baines and Pace, 1991). Nonetheless, other studies could not demonstrate direct coupling between BSP and PER (Teira et al., 2003; López-Sandoval et al., 2010). Thus, beside monomeric compounds, phytoplankton exudates may be composed also of high molecular weight material, mostly polymeric sugars (Myklestad, 1995; Giroldo and Vieira, 2005), which are not directly available for bacteria. In such cases, an enzyme-mediated step is necessary to assimilate these polymeric exudates. The optical properties of PER provide information on PER composition (Stedmon and Markager, 2005; Romera-Castillo et al., 2011). Such investigations, however, do not cover the broad range of non-chromophoric compounds that are also exudated (Rochelle-Newall and Fisher, 2002). Measurements of extracellular enzymatic activity (EEA) is an alternative approach that enables tracking the microbial response to non-chromophoric, but quickly cycling DOM. The activity of extracellular enzymes enables detecting shifts in the microbial response to varying resources (Wagner et al., 2014), especially those that are relatively quickly exploited by the bacterial community. Production of extracellular enzymes by bacteria can be regulated by the DOM supply (Chróst, 1991) and it is directly linked to mechanisms of DOM processing (Sinsabaugh and Foreman, 2001). Hence EEA likely is regulated by the composition of phytoplankton exudates (Chróst and Siuda, 2006).

Model enzymes used to study bacterial degradation of carbohydrates and proteins are β-glucosidase and leu-aminopeptidase (Cunha et al., 2010), but the activity of other enzymes has also been measured frequently (Chróst and Siuda, 2002; Sinsabaugh, 2010). Although some studies report the extracellular enzymatic response to phytoplankton exudates in the water column (Obernosterer and Herndl, 1995; Fajon et al., 1999) and sediments (Goto et al., 2001), this topic remains understudied. Especially under variable hydrological settings in river-floodplain systems this process has been neglected.

Our study was designed to compare water column processes in three different floodplain sections located in a river-floodplain system of the Danube. The focus was on the post-flood period. We investigated relationships between natural phytoplankton productivity and microbial EEA in these distinct subsystems. The main goals of the study were:

- to characterize potential environmental differences between subsystems located in relatively close vicinity in the same floodplain area,
- to investigate how extracellular enzymes respond to primary production, emphasizing the effect of phytoplankton extracellular release in temporarily compared to permanently disconnected water bodies,
- to elucidate the importance of autochthonous, phytoplankton-derived DOM for microbial uptake in an environment typically dominated by allochthonous DOM,
- to elucidate whether the main DOM sources for bacterial growth are different in hydrological individual and diverse subsystems of a floodplain after a flood.

## Materials and methods

### Study site

**Figure 1:**
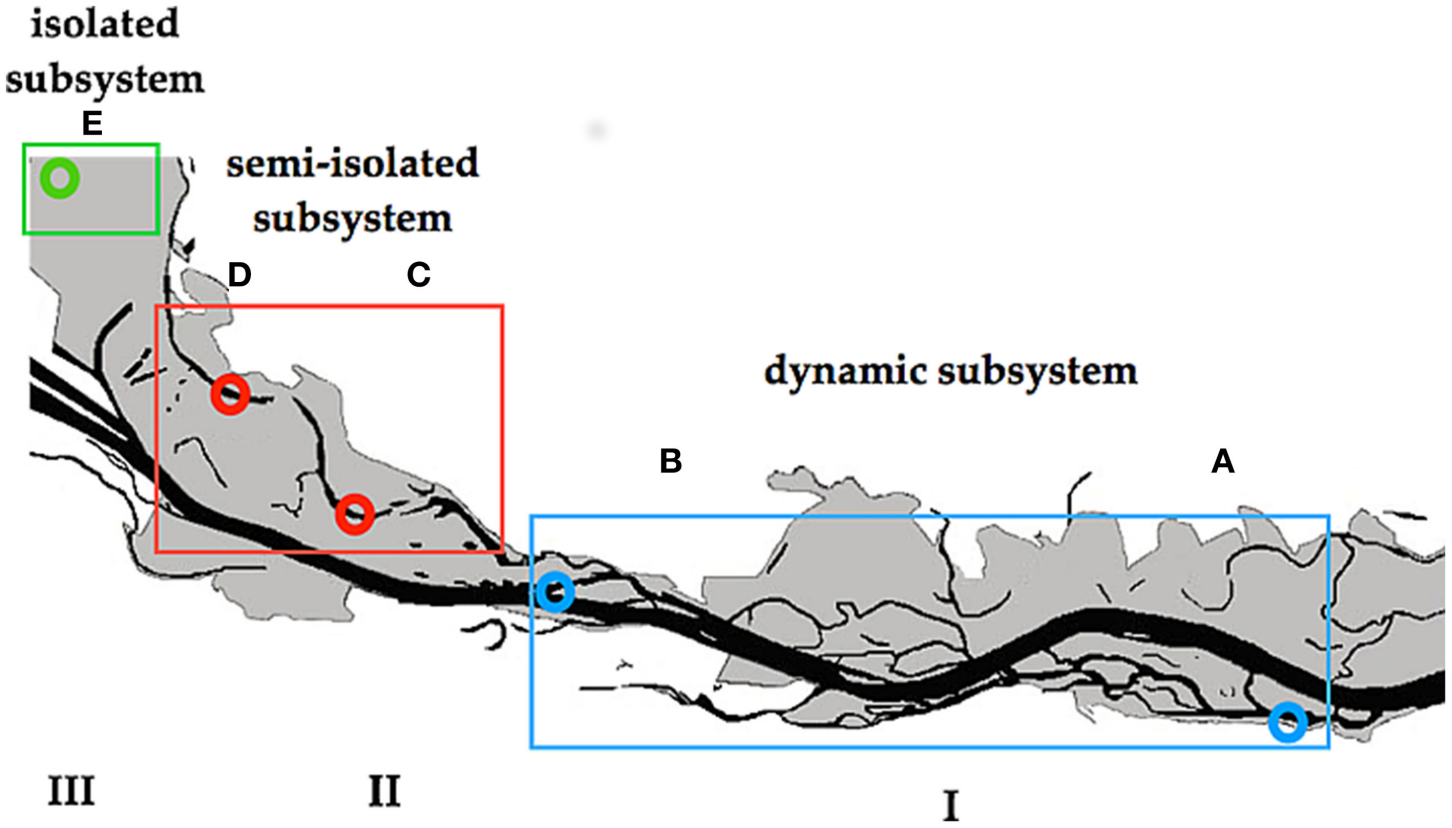
**Danube River and its floodplains downstream of Vienna**. Letters indicate the sampling sites. I–III indicate different subsystems.

The study was conducted in the area of the “Danube Floodplain National Park” (DFNP) downstream of Vienna, Austria. Some parts of this area are severely impacted by human activity (river regulation), whereas others were restored about 15 years ago (Schiemer et al., 1999). Water samples were collected after a 30-year flood from backwaters located in three different subsystems of the DFNP (Figure 1). These subsystems, although close to one another, exhibit distinct hydrological regimes, with different types of surface connectivity with the main Danube channel. Subsystem I (hereafter termed: I) is located in a semi-natural and restored part of the river-floodplain, where two side arms (A and B) were chosen (Figure 1). Although A and B are situated in different sections of the DFNP, both have very similar characteristics. They have been restored by lowering the riverbank, which results in frequent changes of lentic to lotic and flowing conditions (220 (A) and 180 (B) days per year). In subsystem II (hereafter: II) two temporarily disconnected backwaters (C) and (D) were selected (Figure 1). Section C and D are located in a semi-separated area of the floodplain, which is protected from direct flow-through by a levee and additional weirs. Here, a surface connection of backwaters with the main channel is established only rarely (average 18 days per year), during higher water level of the Danube (>1.30 m above mean water). Both backwaters are open water bodies, where DOM derives from external (fallen leaves, rare river water import) as well as from internal sources (phytoplankton, macrophytes). Subsystem III (hereafter: III) in former times was an integral part of the “active” floodplain area, but due to a major regulation of the river in the nineteenth century (embankment and additional weirs), subsystem III has been permanently separated from the main channel. Therefore, it is never flushed even during exceptional floods. Hence, surface connection with the Danube is never established; the only connection with the Danube is through infiltration from groundwater. Subsystem III is situated in a highly shaded area (average light intensity at midday: 195 μmol photons PAR m^−2^ s^−1^) where the dominating DOM input is probably of allochthonous origin (leaves, debris).

Water samples from all stations were always collected between 10 and 12 a.m. with rinsed polyethylene bottles (10 l, single sample) and transported to the laboratory within 3 h in dark boxes at *in situ* temperature. Surface water sampling was performed between July and October 2009, which covered the post-flood period. In subsystems II and III the sampling included a 2-weeks-longer period than in I. Because both locations in subsystem I still experienced flooding conditions in early July, we did not include these samples into our post-flood analyses. Water samples from subsystem I were collected on 5 dates, whereas in II and III, samples were taken on 9 dates. The intensity of sampling was more frequent at the onset of the post-flood phase (every few days) and decreased toward the end of the investigation period (every month). In subsystem I the first sample was taken 7 days after flood while in subsystem II, 3 days after the flooding.

### Analyses of abiotic parameters

Water level, temperature, oxygen saturation (WTW, Oxi 315i) and vertical profiles of light intensity (μmol photons PAR m^−2^ s^−1^, every 10 cm, LICOR sensor) were measured *in situ* at each location. The key nutrients determined included: three nitrogen compounds—nitrate, ammonia, dissolved organic nitrogen (DON)—and soluble reactive phosphorus (SRP). These analyses were completed within 24 h using the German standard methods: nitrate: DIN 38405-29, ammonia: DIN 38406-5, SRP: DIN EN ISO 6678, soluble KjeldahL-N: DIN 38406-5. DON was assessed by subtracting ammonia from soluble KjeldahL-N (Peduzzi et al., 2008) and the ratio DOC/DON was determined. The ratio of dissolved inorganic nitrogen [(nitrate+ammonia) (DIN)] and SRP was calculated as DIN/SRP.

### Primary and secondary producers

#### Phytoplankton productivity and biomass

To make our measurements comparable, the samples for primary production were taken at the same time of day during all sampling events right below the water surface (10–20 cm). Particulate primary production (pPP) and net photosynthetic extracellular release (PER) of phytoplankton were determined using a ^14^C method, originally proposed by Steemann-Nielsen (1974), modified according to Preiner et al. (2008). We measured the rate of carbon fixed in particulate material (cells; pPP), while PER was assessed to estimate dissolved organic carbon released by phytoplankton into the surrounding water. Total primary production (PPt) was calculated as the sum of pPP and PER. Assessing PPt was needed to calculate the relative fractions. For each station, 8 bottles with a defined volume (~117 ml) were incubated in a water basin at *in situ* temperature for 3.5–4 h after adding sodium bicarbonate (NaH^14^CO_3_) ^14^C (20 μCi/ml). This time period was selected according to Myklestad (2000) and Preiner et al. (2008) assuming that, on one hand, it was not too short to obtain measurable results. On the other hand, avoiding that the bacterial community has not passed its latent period and not reached maximum uptake rates. With this it was attempted to minimize a bias due to potentially remaining bacterial uptake. Bottles were exposed in a light gradient covering the environmental conditions (10–560 μmol Photons m^−2^ s^−1^). The sample water was permanently mixed by placing the bottles on a rolling table. Two additional bottles were kept in dark to determine the unspecific adsorption of ^14^C. After the incubation, filtration (0.45 μm; Millipore HAWP) separated the particulate from the dissolved fraction (Wetzel and Linkens, 1990) using low vacuum pressure (<50 mBa). The filtrate from each sample (5 ml), including dark-incubated samples, was collected. The filters were placed in scintillation vials with 0.5 ml 0.5N HCl to remove the inorganic ^14^C (Peterson, 1980). The filtrate was decontaminated by acidifying the samples to pH 4 and bubbling with air for 15 min (Peterson, 1980; Parsons et al., 1984). Filters were dissolved with ethyl acetate to minimize self-adsorption (Peterson, 1980) and scintillation cocktail was added. After 24 h radioactivity of each sample was measured in a scintillation counter (Parsons et al., 1984) to determine disintegrations per minute (DPMs). DPMs of dark-incubated samples were subtracted to calculate pPP and PER. For the characterization of the light climate, the light attenuation coefficient was determined from vertical light profiles and light curves (P/I curves) were created. Primary production [μg C l^−1^ h^−1^] was calculated according to Jassby and Platt (1976) based on surface radiation, the attenuation coefficient and the depth where the samples were taken (Riedler and Schagerl, 1998). The analytical variability was always <10%. The rate of carbon fixed in particulate organic matter (cells; pPP) was compared to the rate of dissolved organic carbon released extracellularly by phytoplankton (PER). The relative extracellular release was defined as percentage of total primary productivity (PPt): %PER = PER × 100/(PP+PER). Additionally, for a biomass estimate, the concentrations of chlorophyll *a* (chl *a*) were determined using standard methodology (Lorenzen, 1967); the method is described in more details by Schagerl et al. (1996).

#### Bacterial secondary production

Bacterial secondary production (BSP) was assessed based on the method of [^3^H] thymidine (20 μl, 20 nM final concentration) incorporation into DNA (Fuhrman and Azam, 1982). Three replicate samples and two additional formaldehyde-killed (2% final concentration) samples (5 ml), which served as controls, were incubated in dark at ambient temperature for 0.45–1.5 h. After the incubation, samples and controls were filtered (0.45 μm; Millipore HAWP). Filters were collected in scintillation vials and dissolved in ethyl acetate. Afterwards scintillation cocktail was added and samples were placed into the scintillation counter. The analytical variability was always <10%. BSP [μg C l^−1^ h^−1^] was calculated using an average Danube-specific conversion factor of 3.2 × 10^18^ cells produced per mol of incorporated thymidine (Berger et al., 1995). The ratio of BSP:PPt was calculated, based on hourly rates as an instantaneous BSP/PPt ratio, the ratio right at the day time of sampling. This ratio was used to establish the relative share of primary production used by BSP, hence to characterize a potential coupling between the bacterial and phytoplankton compartments (Ducklow and Carlson, 1992).

#### Bacterial abundance

Sample water (1–2 ml) was fixed with formaldehyde (2% final concentration) and stained with 4.6-diamidino-2-phenylindole-dihydrochloride (DAPI) (10 μg ml^−1^). Bacterial abundance (BA) was determined by epifluorescence microscopy (Nikon E 800) in 20–30 randomly chosen fields according to Porter and Feig (1980).

#### DOM characterization

Water samples were filtered through pre-combusted (500°C) GF/F filters (0.7 μm, pore size) within 3 h of collection. Samples for DOC concentration [mg l^−1^] were acidified to pH 3 and analyzed by high-temperature combustion using a Shimadzu TOC 5000 analyzer (Benner and Strom, 1993). We applied optical DOM indices to obtain information about DOM quality in each subsystem. Absorbance at wavelengths of 364, 254, and 250 nm was measured in a 5-cm quartz cuvette with a spectrophotometer (Hitachi U-2000). Carbon-specific UV absorbance (SUVA_254_) [l mg^−1^ m^−1^] was calculated as the ratio of absorbance at 254 nm to the DOC concentration. SUVA_254_ is reported to be positively correlated with DOC aromaticity (Weishaar et al., 2003). SUVA_254_ has also been used as an indicator of terrestrial sources of DOM (Jaffé et al., 2008). Additionally, the ratio of absorbance measured at 250 nm to absorbance at 364 nm (E2:E3 ratio), first proposed by De Haan and Boer (1987), was calculated. The E2:E3 ratio was determined to track changes in the relative size of DOM molecules. E2:E3 is inversely related to average DOM molecular weight, hence higher E2:E3 ratios, indicate lower molecular weight of DOM. The E2:E3 ratio has been shown to be a good indicator to track alterations in molecular weight of DOM during and after rapid hydrological changes (Ågren et al., 2008). Furthermore, to distinguish DOM originating from microbial sources (including algal-derived) from terrestrially derived DOM, the fluorescence index (FI) (McKnight et al., 2001; Sieczko and Peduzzi, 2014) was determined with a Shimadzu spectrofluorophotometer RF-5301 PC. FI is the ratio of the emission intensity at 450–500 nm under excitation at 370 nm; FI end-values of ~1.3 indicate allochthonously produced DOM, whereas ~2.0 implies autochthonously derived material.

#### Extracellular enzymatic activity (EEA)

EEA was measured to infer available organic matter sources as suggested by Boschker and Cappenberg (1998) for natural systems. Hence we used EEA as a tool to obtain information about availability of non-chromophoric but reactive and quickly cycling DOM (Sieczko and Peduzzi, 2014). Part of the rapidly metabolized DOM (including phytoplankton-derived DOM) is non-chromophoric, but it also includes bioavailable polysaccharides and proteins. Hence, this part of the DOM pool cannot be tracked with standard optical methods. Therefore, serving as a proxy, this ecologically important DOM was investigated by EEA measurements.

Fluorogenic substrate analogs were used to assess potential hydrolysis rates of α-, β- glucosidic and peptide bonds. To estimate the activity of α- (EEAa) and β- (EEAb) glucosidase, 4- methylumbelliferyl (MUF)-α-D-glucoside and 4-MUF-β-D-glucoside were used, respectively. To measure the activity of leucine aminopeptidase (EEAleu), L-leucine 7-amido-4-methylcoumarin was used. For measuring the extracellular enzymatic activity, triplicates of 3 ml of sample water with 15 μl fluorescently- labeled substrates (final, substrate saturating concentration: 2.5 μM) were incubated in the dark at *in situ* temperature. Fluorescence of the replicates was measured immediately after adding the substrate (t_0_) and after 45–90 min (t_1_) at an excitation wavelength of 360 nm and an emission wavelength of 444 nm (Hoppe, 1983) with a spectrofluorophotometer (Shimadzu RF-5301 PC). Substrate degradation was calculated from the increase in fluorescence over time as nmol substrate hydrolyzed per liter per hour (Hoppe, 1993).

Phenol oxidase activity (PhOx) was measured spectrophotometrically following the method outlined by Pind et al. (1994). Incubations were completed by mixing 2 ml of unfiltered sample water with 2 ml of L-3,4-Dihydroxyphenylalanine (DOPA) stock solution (5 mM DOPA in 2.5 mM NaHCO_3_ buffer, pH 8.3). Thus, the substrate saturating, final concentration for our samples was 2.5 mM DOPA. The absorbance was measured at 460 nm in a spectrophotometer (Hitachi U-2000) immediately after addition of DOPA (t_0_) and after incubation in dark (t_1_) at *in situ* temperature for 180–220 min. Results were calculated using Beer's Law and the molar absorbancy coefficient for the DOPA product 3-dihydroindole-5.6-quinone-2-carboxylate (diqc) (3.7 × 10^4^; Mason, 1948). Phenol oxidase activity was expressed in nmol of product (diqc) produced per liter per hour.

#### Statistical analyses

For the statistical tests we used R 2.15.0 and SPSS 17.0 for Windows. Most of the data were normally distributed and fulfilled the conditions to apply parametric tests (*T*-test and One-Way ANOVA), unless stated otherwise. In such cases, non-parametric tests such as Wilcox- and KruskaL-Wallis tests were applied. Only the data used for correlations and linear regressions were log-transformed and the residuals were tested for normal distribution and homogeneity of variances.

## Results

### Selected environmental and DOM characteristics

Most of the abiotic parameters of subsystem III were clearly distinct from parameters at subsystems I and II. Different nutrient conditions occurred: in subsystems I and II, SRP was significantly lower compared to III, (*p* < 0.001, *n* = 42), whereas the lowest DIN was noted in II. Hence the DIN/SRP-ratio diverged from the Redfield ratio and was significantly higher in I and II than in III (*p* < 0.001, *n* = 42) (Table 1). Average oxygen saturation [O_2_%] was significantly different between all subsystems (*p* < 0.001, *n* = 42), with the highest values at I (Table 1). The organic nutrient ratio (DOC/DON) ranged from 13:1 to 62:1 (Table 1), with significantly higher values at II and III compared to I (*p* < 0.001, *n* = 42). The DOC quantity and the qualitative proxies for DOM in the permanently disconnected subsystem (III) were distinct from I and II, with significantly higher DOC (*p* < 0.001), SUVA_254_ (*p* < 0.01), higher E2:E3 ratio (*p* < 0.05) and lower FI (*p* < 0.001) in III (Table 1). The DOC quantity in I and II decreased by 50% during the investigation period, while in III it increased by 30%, (data not shown). Also SUVA_254_ in I and II dropped (43, 57%, respectively) while E2:E3 increased by 20 and 35%, respectively, toward the end of the sampling season (early October) compared with the values measured at the onset of the post-flood period. DOM quantity and quality in subsystems I and II did not significantly differ from each other. In III SUVA_254_ and E2:E3 were relatively constant; the only considerable increase was noted in the middle of the sampling season (data not shown).

**Table 1:** **Min-max values of: nutrients (DIN, SRP, DIN/SRP ratio), oxygen saturation and DOM quantity (DOC) and quality (DOC/DON, SUVA_254_, FI ratio, E2:E3 ratio) in subsystems I–III; average in brackets**.

**Subsystem**	**DIN [μg l^−1^]**	**SRP [μg l^−1^]**	**Ratio DIN/SRP [g/g]**	**O_2_ [%]**	**DOC [mg l^−1^]**	**Ratio DOC/DON**	**SUVA [l mg^−1^ m^−2^]**	**FI**	**E2:E3 ratio**
I	107.5–1478.9	0.88–25.7	37.3–485.1	74–147	1.30–2.61	12.9–22.9	3.53–7.53	1.52–1.68	4.66–6.79
	(743.1)	(8.68)	(181.7)	(104)	(1.88)	(17.1)	(5.79)	(1.62)	(5.82)
II	86.9–349.7	0.78–3.24	60.7–332.5	50–100	1.98–4.37	13.3–41.5	3.58–8.38	1.40–1.66	4.34–6.69
	(181.4)	(1.44)	(132.8)	(75)	(2.63)	(23.2)	(6.34)	(1.58)	(5.34)
III	368.7–1051.8	121.7–581.4	1.05–4.47	10–65	21.21–389.0	17.4–62.4	5.60–12.9	1.42–1.53	4.88–10.7
	(771.8)	(364.9)	(2.41)	(38)	(27.0)	(25.6)	(7.94)	(1.51)	(6.26)

*For abbreviations see text*.

### Phytoplankton biomass, primary production and photosynthetic extracellular release

Phytoplankton biomass and productivity varied distinctly along the connectivity gradient from dynamic to isolated sites. After the flood, in mid-August, the pPP increased markedly and reached the highest values in station A (263.4 μg C l^−1^ h^−1^) and B (213.1 μg C l^−1^ h^−1^) (Supplementary Figures 1A,B). In subsystem II, the pPP also increased after the flood, the highest pPP rate 181.9 μg C l^−1^ h^−1^ occurring at the end of the sampling period (Supplementary Figures 1C,D). In subsystem III, pPP was much lower and fluctuated considerably, peaking in September (41.9 μg C l^−1^ h^−1^) (Supplementary Figure 1E).

**Figure 2:**
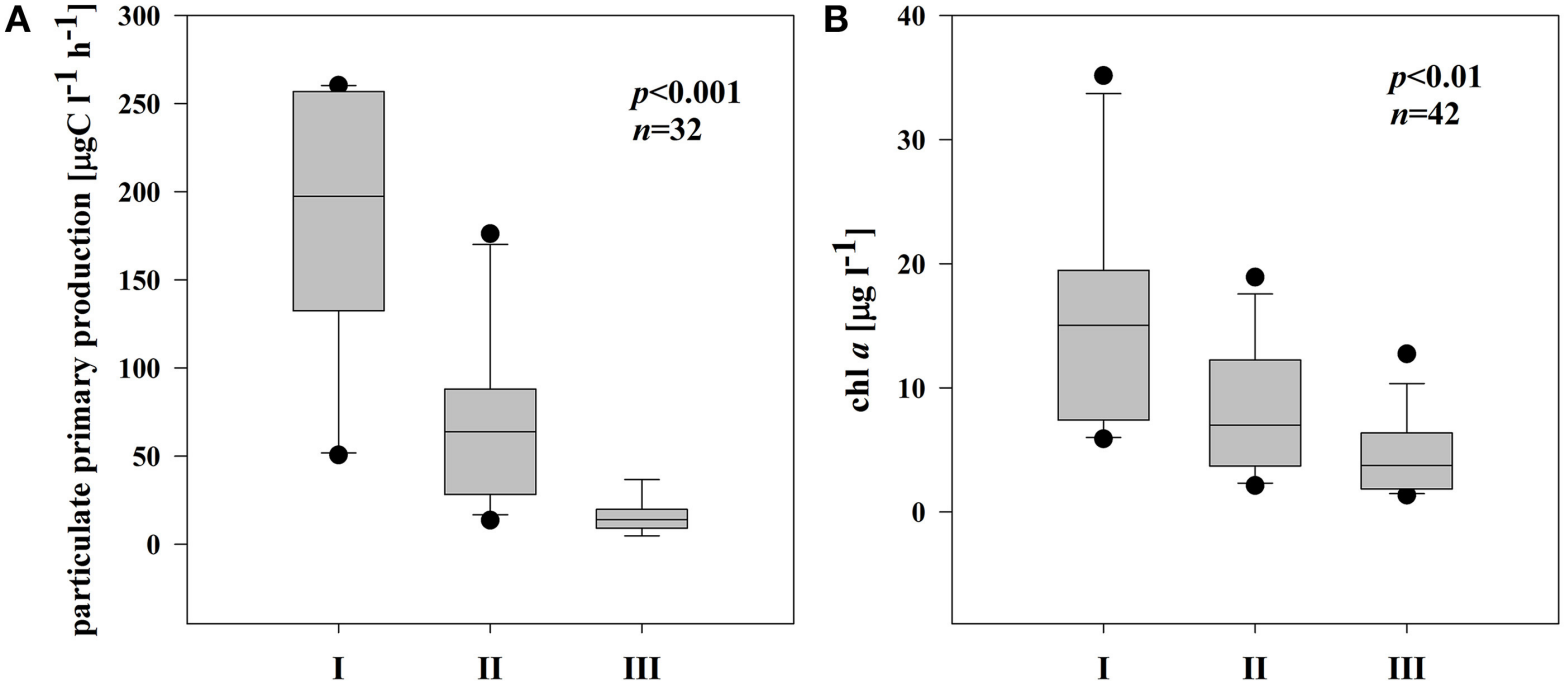
**Boxplots illustrating average chl *a* (A) and pPP rates (B) in three different subsystems (I–III) during the sampling period**. The boundaries of the box plot indicate the 25th and 75th percentiles, points indicate outliers, the solid line in the box marks the median. Statistical differences are indicated on the top of each panel (for applied statistical tests see Section Materials and Methods).

On average, chl *a* and pPP rates were highest in I; pPP rates in III were even 7 times lower than in I (Figures 2A,B). Chl *a* and pPP were correlated significantly only in the semi-isolated subsystem (II) (*r* = 0.82, *p* < 0.001, *n* = 14).

**Figure 3:**
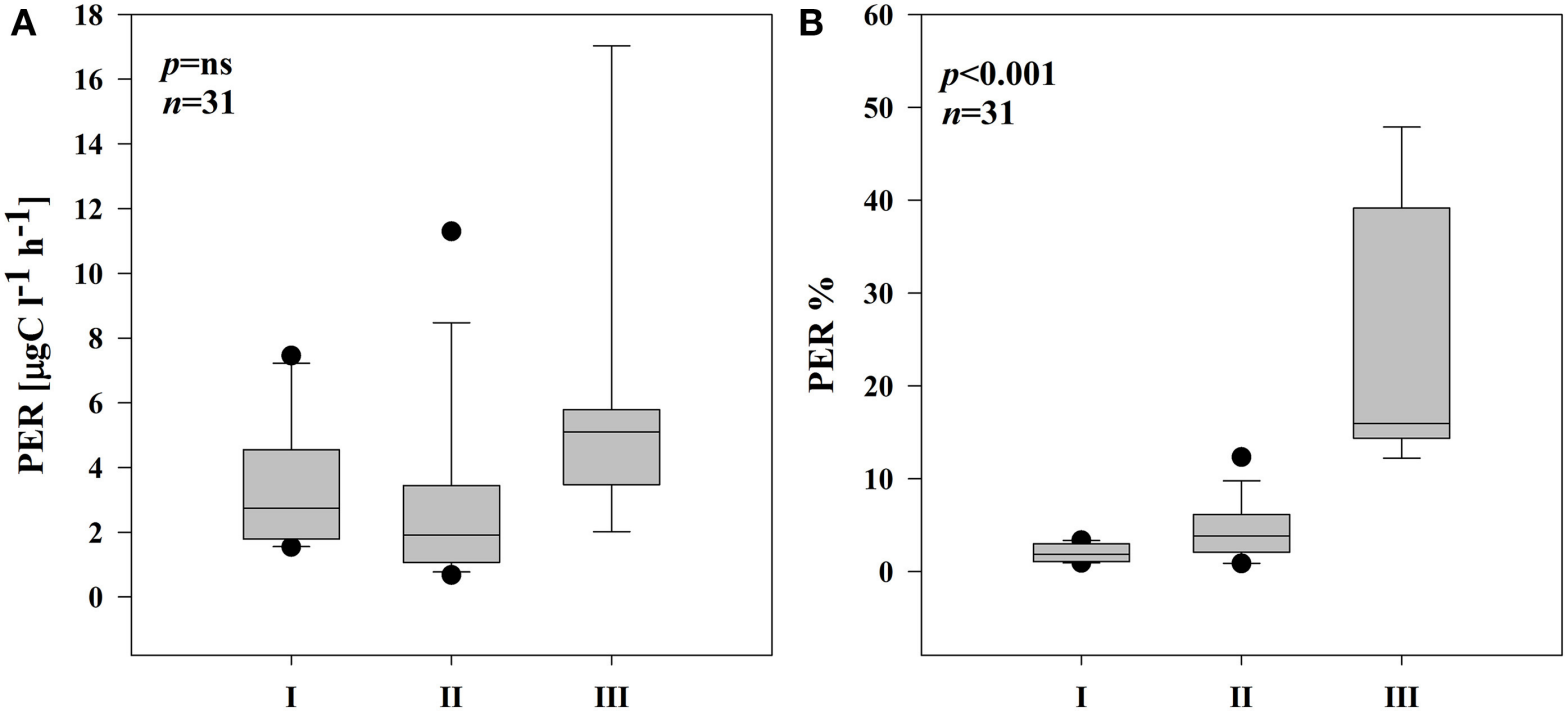
**Boxplots illustrating average PER rate (A) and % of PER release in PPt (B) in three different subsystems (I–III) during the sampling period**. The boundaries of the box plot indicate the 25th and 75th percentiles, points indicate outliers, the solid line in the box marks the median. Statistical differences are indicated on the top of each panel (for applied statistical tests see Section Materials and Methods).

Phytoplankton cells excreted variable amounts of photosynthesis products (PER) into the water. PER in I remained in the range of 1.54–7.46 μg C l^−1^ h^−1^ (Supplementary Figures 2A,B). The highest PER coincided well with an increase in pPP rates in both locations in I. In II and III, PER fluctuated considerably (0.68–11.3 μg C l^−1^ h^−1^ and 2.02–17.0 μg C l^−1^ h^−1^, respectively) with the highest rates in the end of the sampling period (Supplementary Figures 2C–E). Overall, subsystem III was characterized by generally higher rates of PER, but due to high variability, no significant differences in PER between the subsystems were noted (Figure 3A). Also the percentage of PER (% PER) was highly variable in each subsystem throughout the sampling season. Nonetheless, average % PER increased significantly at locations with lower connectivity with the main river channel (Figure 3B). The share of PER on total primary production (PPt) in I ranged from 0.9 to 3.4%, in II from 0.9 to 12.3% whereas in the isolated subsystem (III), the values were between 12.2 and 47.9% of PPt.

### Extracellular enzymatic activity in different subsystems of the floodplain

**Figure 4:**
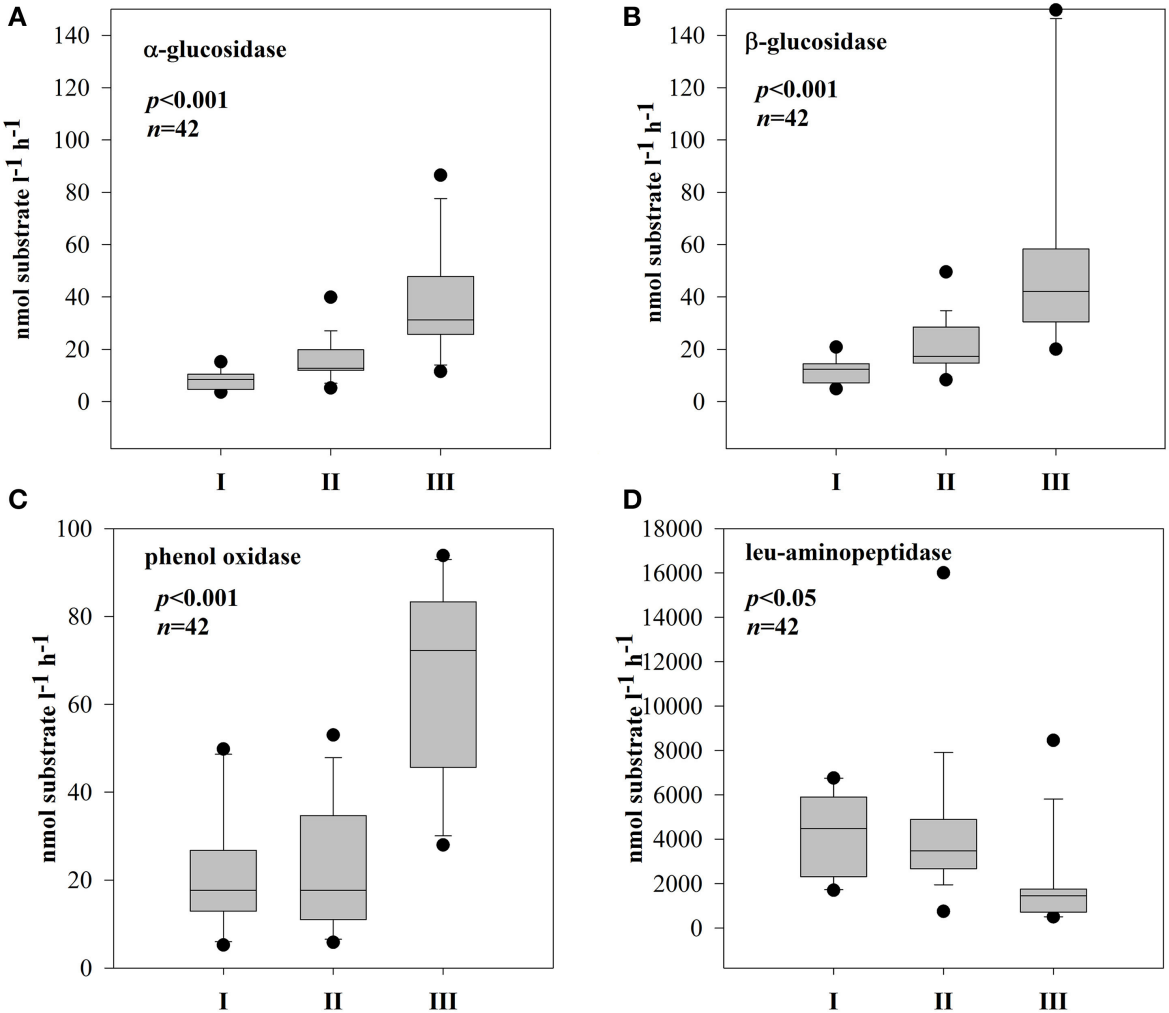
**Boxplots of extracellular enzymatic activity of α-glucosidase (A), β-glucosidase (B), phenol oxidase (C), and leu-aminopeptidase (D) in three different subsystems (I–III)**. The boundaries of the box plot indicate the 25th and 75th percentiles, points indicate outliers, the solid line in the box marks the median. Statistical differences are indicated on the top of each panel (for applied statistical tests see Section Materials and Methods).

The EEA rates showed different patterns in the three subsystems (Supplementary Figure 2). In both stations of subsystem I, all the measured enzymes strongly coincided with each other (Supplementary Figures 2A,B); hence, hydrolases and oxidase were strongly and positively correlated (Table 2A). In subsystem II, EEA was more variable, but, the hydrolytic enzymes coincided quite well during the investigated post-flood period (Supplementary Figures 2C,D); correlations between hydrolytic enzymes (glucosidases with leu-aminopeptidase) were still observed (Table 2B). The hydrolase activity peaked in subsystem III, while PhOx showed the lowest rates here toward the end of the sampling period (Supplementary Material Figure 2E). Extracellular enzymes in III were more decoupled, but similarly to other subsystems the activities of the two glucosidases (EEAa and EEAb) coincided well throughout the investigated post-flood period (Table 2C). Activity of glucosidases (Figures 4A,B) and PhOx (Figure 4C) were on average highest in III, whereas EEAleu was highest in the dynamic subsystem (I) (Figure 4D). Among the hydrolytic enzymes, EEAleu displayed the highest activity, which was 3–4 orders of magnitude higher than EEAa and EEAb activity. In line with the gradient of connectivity between I, II, and III, decoupling of the different enzymatic activities occurred (Table 2).

**Table 2:** **Correlation coefficients between extracellular enzymatic activity (EEAa, EEAb, EEAleu, phOx) in subsystem I (A), subsystem II (B), and subsystem III (C)**.

	**EEAa**	**EEAb**	**EEAleu**	**phOx**
**I (A)**				
EEAa	–	0.98^***^	0.86^**^	0.73^*^
EEAb		–	0.90^***^	0.70^*^
EEAleu			–	0.76^*^
phOx				–
**II (B)**				
EEAa	–	0.98^***^	0.50^*^	ns
EEAb		–	0.54^*^	ns
EEAleu			–	ns
phOx				–
**III (C)**				
EEAa	–	0.74^*^	ns	ns
EEAb		–	ns	−0.65^*^
EEAleu			–	ns
phOx				–

* *p < 0.05*,

** *p < 0.01*,

*** *p < 0.001. The data were log-transformed*.

### Bacterial abundance and production

**Figure 5:**
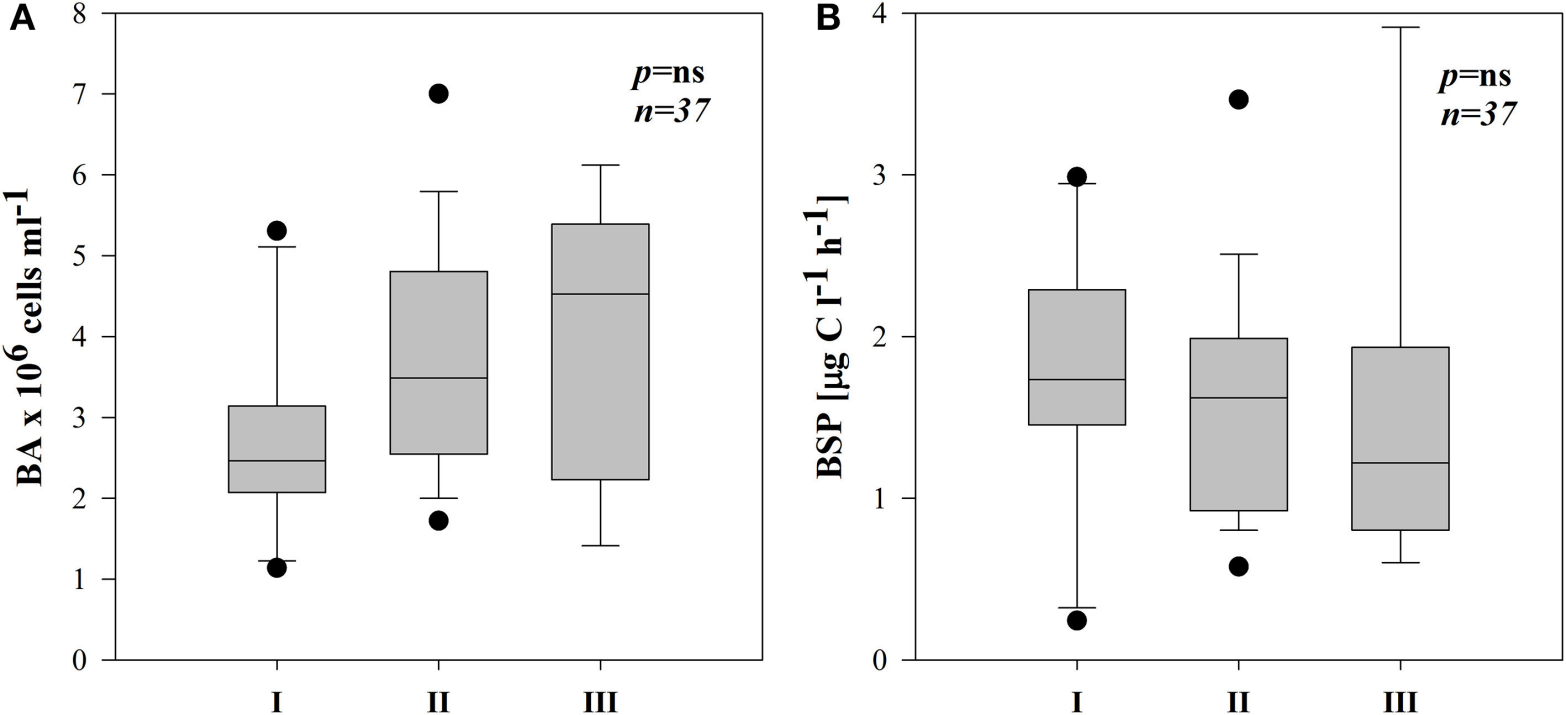
**Boxplots of average BA (A) and BSP (B) in three different subsystems (I–III)**. The boundaries of the box plot indicate the 25th and 75th percentiles, points indicate outliers, the solid line in the box marks the median. Statistical differences are indicated on the top of each panel (for applied statistical tests see Materials and Methods).

Total bacterial numbers (BA) in all the subsystems ranged from 1.1 to 7.0 × 10^6^ cells ml^−1^ (Supplementary Figures 3A–E). They were on average higher in III but not significantly different between the three subsystems (Figure 5A).

BSP ranged from 0.24–3.91 μg C l^−1^ h^−1^ with occasionally highest rates in III (Supplementary Figures 3A–E). Due to high variability, however there were no significant differences between subsystems (Figure 5B). BSP followed a similar pattern as BA in subsystems I and II (Supplementary Figures 3A–D), hence BSP was significantly correlated with BA in I (*r* = 0.82, *p* < 0.01, *n* = 10) and in II (*r* = 0.56, *p* < 0.05, *n* = 18).

### Microbial response on DOM characteristics

**Figure 6:**
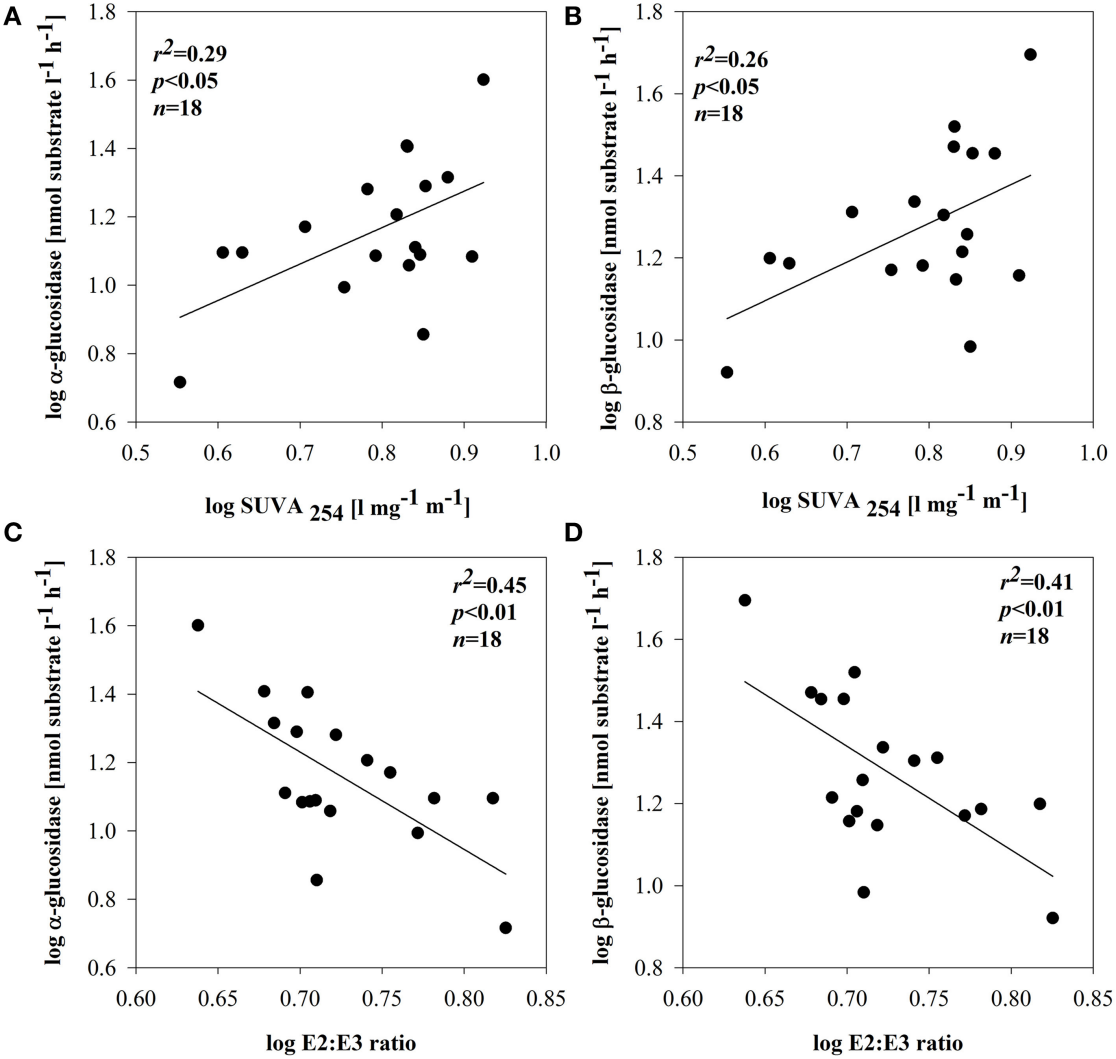
**Relationships between SUVA_254_ and: α-glucosidase (A) or β-glucosidase (B) and E2:E3 ratio and: α-glucosidase (C) or β-glucosidase (D) in subsystem II**. All data were log-transformed.

BSP and BA were not related to DOM quality (based on optical properties) in any of the subsystems. No direct enzymatic response to any of the qualitative proxies was observed in subsystem I and III. In subsystem II, however, the synthesis of EEAa and EEAb was apparently related with SUVA_254_ (Figures 6A,B). EEAa and EEAb were also negatively related to the E2:E3 ratio (Figures 6C,D).

### Relationships between extracellular enzymatic activity and primary and secondary production

**Figure 7:**
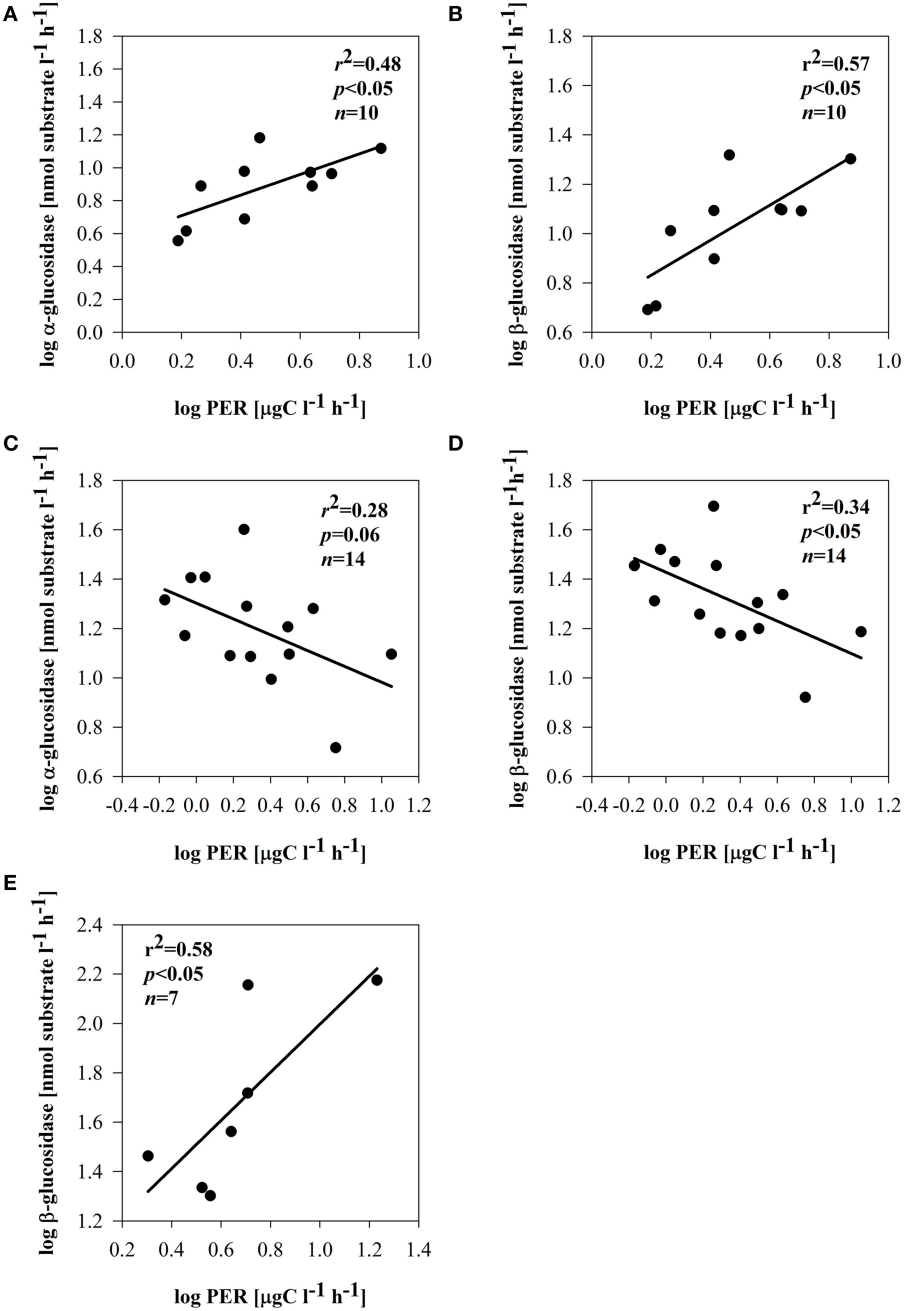
**Relationships between PER and α-glucosidase and PER and β-glucosidase in subsystem I (A,B), subsystem II (C,D), and subsystem III (E)**. All data were log-transformed.

Depending on the subsystem, the extracellular enzymes exhibited distinct relationships with phytoplankton and bacterial parameters. In the dynamic subsystem (I), hydrolase activity was strongly promoted by the primary production rate and biomass (pPP and chl *a*) (Table 3). Beyond a positive relation between PhOx and chl *a* (*r* = 0.46, *p* = 0.05, *n* = 14) in II, no other direct enzymatic response to chl *a* occurred in subsystems II and III. There was, however, a clear enzymatic response to phytoplankton extracellular release. The synthesis of α -and β-glucosidase clearly coincided with PER in subsystem I (Supplementary Figures 2A,B); EEAa and EEAb were apparently promoted by PER (Figures 7A,B). However, in subsystem II, the opposite tendency was noted; the activity of EEAa and EEAb was negatively related to PER (Figures 7C,D). In subsystem III, EEAb corresponded to PER (Supplementary Material Figure 2E), hence it was positively related to PER (Figure 7E).

**Table 3:** **Correlation coefficients between extracellular enzymatic activity (EEAa, EEAb, EEAleu, PhOx) and phytoplankton parameters (pPP and chl *a*) in subsystem I**.

	**pPP**	**chl *a***
EEEa	0.82^**^	0.81^**^
EEAb	0.85^**^	0.87^**^
EEAleu	0.83^**^	0.77^**^
phOx	ns	ns

** *p < 0.01. The data were log-transformed*.

Since DOM properties, based on our optical assessment of DOM quality, were not related to BSP or BA, the enzymatic approach helped to elucidate which DOM was important for bacterial metabolism in each subsystem. Accordingly, we linked EEA to BSP to investigate which DOM pool was more important for bacterial metabolism in our three different subsystems. Our results imply that degradation of distinct material supported bacterial growth in each subsystem differently. More than one enzyme explained BSP variability; we therefore performed stepwise multiple regression analyses using a combination of EEAleu, EEAa, EEAb, and PhOx as independent variables. This revealed that in each subsystem different enzymes were the most significant predictors for BSP (Table 4). In I, only EEAleu was directly related to bacterial production. In II, BSP was best described by the activity of EEAleu and PhOx, while in subsystem III, EEAb explained most of the variability in BSP (Table 4).

**Table 4:** **Multiple stepwise regressions between bacterial secondary production (BSP) and enzymatic activity (EEAa, EEAb, EEAleu, PhOx)**.

	**Independent variables**	**Regression equation**	**Adjusted r^2^**
Subsystem I BSP	EEAleu(beta = 0.65; *p* < 0.05)	logBSP = 2.527+0.895 logEEAleu	0.35
Subsystem II BSP	PhOx(beta = 0.56; *p* < 0.01)	logBSP = 0.062+0.397 logPhOx	0.60
	EEAleu(beta = 0.45; *p* < 0.05)	logBSP = −1.080+0.342 logEAleu	
Subsystem III BSP	EEAb(beta = 0.86; *p* < 0.001)	logBSP = −0.469+0.797 logEEAb	0.72

*All data were log transformed. For abbreviations see text*.

In I, pPP was directly related to BSP (*r* = 0.67, *p* < 0.05) and BA (*r* = 0.64, *p* < 0.05). Phytoplankton biomass (chl *a*) also positively related to BA in I (*r* = 0.65, *p* < 0.05) and BSP in II (*r* = 0.49, *p* < 0.05, *n* = 17). There was no direct response of bacterial production or abundance to photosynthetic extracellular release (PER) in any of the subsystems.

In subsystem I, where the highest pPP was observed (Figure 3), the BSP:PPt ratio was the lowest; bacterial production there ranged from 0.38 to 3.06% (average 1.07%) of the total primary production. On average the BSP:PPt ratio was more than 2 times higher in III than in II, where it remained in a range of 2.01–17.7% (8.65%) and 0.58–10.6% (3.66%), respectively.

## Discussion

### Enzymatic activity: response to DOM quality

This study was designed to shed light on the microbial response to various allochthonous and autochthonous DOM sources in different sections of a floodplain. Our results show that DOM in all subsystems was a mixture of allochthonous and autochthonous material. Although no significant differences were noted between subsystem I and II after the flood, this study points to the more autochthonous, less aromatic DOM in subsystem I. However, in subsystem III DOM was clearly more allochthonous, higher molecular weight and more aromatic. This is very likely due to input of local terrestrial material (dense surrounding vegetation), because this subsystem is never connected to the main channel. In other subsystems (I and II) allochthonous material derives also from the main channel during occasional surface connection with the Danube (Sieczko and Peduzzi, 2014). Differences in the DOM composition and availability can be important in determining the functioning of microbial communities (Hoostal and Bouzat, 2008). Our results, however, do not generally support the idea that enzymatic activity is strongly linked to DOC quantity or quality based solely on optical measurements. Even though the quantitative and qualitative parameters of DOM were significantly different between the investigated subsystems, only in subsystem II did we find a significant relation between enzymatic activity and optical properties of DOM.

The positive relation between EEAa, EEAb activity and SUVA_254_ or molecular size of DOM (Figures 6A–D) implies enhanced expression of glucosidases in the presence of terrestrially derived, higher molecular weight DOM. There is evidence that HMW substances can be even more reactive as long as they are diagenetically younger (Amon and Benner, 1996) compared to diagenetically older LMW residues of former bacterial metabolism (Ogawa et al., 2001). Nonetheless, molecular weight or origin are not the only factors regulating DOM bioavailability. For example, microbial communities are most productive when metabolizing their native DOM sources (Young et al., 2005). This could explain that, in other subsystems, EEA was not related to the molecular size of DOM and that other factors might have been important.

### Relationship between phytoplankton and microbial enzyme activities

The mechanisms behind enzyme synthesis are regulated by a number of environmental factors which may induce or suppress production of extracellular enzymes (Sinsabaugh and Follstad Shah, 2012; Arnosti et al., 2014). In our study, we attempted to evaluate the importance of autotrophic planktonic production for EEA. Our results show that in subsystem I, primary production (pPP and chl *a*) was strongly and positively related to EEAleu (Table 1), which indicates the importance of algal products here. Studies in river environments showed that EEAleu was actively produced when autochthonous production of labile organic matter was high (Wilczek et al., 2005). One potential reason for quite high activity levels of EEAleu in our study, compared to the activity of other enzymes, is that aminopeptidases are not very specific in their cleaving (Chróst, 1992) and that they can hydrolyze many different peptides.

In I we also observed strong correlations between all the enzymes, including PhOx (Table 3). This indicates a tight coupling of metabolic pathways (Hoostal and Bouzat, 2008) and mutualism within the microbial community here. Further, in subsystem II, the strong and positive relation between PhOx and chl *a* may indicate that input of fresh labile material from primary producers was able to augment synthesis of PhOx.

The phenomenon of enhanced production of extracellular enzymes degrading recalcitrant OM in the presence of labile OM is called priming effect (PE) (Guenet et al., 2010; Bianchi, 2012) and it has already been proven in laboratory assays (Shimp and Pfaender, 1985) and field studies (Treignier et al., 2006; Rier et al., 2007, 2014). Although our data may imply that in subsystems I and II phytoplankton-derived material possibly could influence the expression enzymes responsible for degrading recalcitrant DOM, more extensive studies on a potential priming effect here would be necessary. However, we suggest that river-floodplain systems are places where ample opportunities for this phenomenon are created. This calls for further investigations on the existence of a priming effect in hydrologically dynamic river-floodplain systems.

Carbon-rich exudates are often predominant products of phytoplankton growing under an imbalanced DIN/SRP ratio (Penna et al., 1999). Production of bacterial enzymes may be also related to the nutrient status. We propose that strong P-limitation (and low SRP) noted in subsystem II (Table 1) may have induced C-rich PER. Under P-depleted conditions, phytoplankton releases large amounts of carbohydrates (Obernosterer and Herndl, 1995), which may have induced a high utilization of PER by bacteria. Utilizable PER implies a reduction of EEAb activity, and this could be responsible for the negative relation of glucosidases with PER (Figures 7C,D). A decrease of β-glucosidase produced by aquatic bacteria has been described as being linked to the introduction of phytoplankton-derived DOM (Chróst and Siuda, 2002). Furthermore, also in lake water the catabolic repression of glucosidases in the presence of easily assimilable substrates is described as an important mechanism controlling EEAb synthesis (Chróst, 1989).

However, in subsystem III nutrient limitation was unlikely, because DIN and SRP were high (Table 1). Other reasons (strong light limitation or viral lysis), potentially responsible for elevated PER here, need to be considered (compare Myklestad, 2000). In subsystem III (Table 1), a large portion of total primary production was exudated, reaching up to 50% (Figure 3B). The positive relation between PER and EEAb (Figure 7E) in subsystem III implies utilization of phytoplankton exudates that are mainly comprised of β-linked polysaccharides; these are often a predominant product of phytoplankton extracellular release (Biddanda and Benner, 1997) and from lysed cells (Weinbauer and Peduzzi, 1995). Although some of the primary production exudates contain quickly consumed compounds, actively growing phytoplankton also releases higher molecular weight organic matter, which is largely composed of polysaccharides (Lignell, 1990; Myklestad, 2000). We suggest that the quality of exudates triggered a greater activity of EEAb.

### What supports bacterial production in distinct floodplain subsystems?

Based on our study, EEA appears to be a valuable tool for elucidating the potential linkage between non-chromophoric, but rapidly cycling DOM and bacterial production. Our results imply that in each of the three subsystems, the degradation of distinct material supported bacterial growth. The similar average BA and BSP rates in all subsystems (Figures 5A,B)—but significantly different EEA (Figures 4A–D), phytoplankton biomass and pPP rates (Figures 2A,B)—suggest that in each of the three subsystems, bacterial growth was supported by DOM from different sources. Typically, DOM of autochthonous origin is higher-quality material that mainly controls BSP (Cole et al., 2002), as opposed to allochthonously derived DOM, which is recalcitrant with much longer turnover times. These pools are produced independently from each other but both are available for bacteria (Del Giorgio and Pace, 2008). Although correlation between BSP and pPP has been demonstrated both in culture (Gurung et al., 1999) and field studies (Cole et al., 1988), our field data show that only in subsystem I was primary production (pPP) directly coupled to BSP or BA (see Results). At increased primary productivity bacteria can assimilate more phytoplankton-derived DOM (Piontek et al., 2012). Hence, we suggest that in subsystem I (with highest pPP noted), pPP derived-material supported bacterial production directly or by an enzyme-mediated step. Other studies from this area (Preiner et al., 2008) report stimulated phytoplankton productivity after floods in frequently connected water bodies. Also, according to the River Wave Concept (Humphries et al., 2014), autochthonous production of DOM predominates after floods. Hence, strong relation of pPP with EEAleu (Table 3) and of BSP with EEAleu (Table 4) implies that pPP products, hydrolyzed by EEAleu, supported BSP in I. Our results suggest that in frequently flooded river-floodplain systems (such as subsystem I), elevated bacterial growth could be driven mainly by enzymatic degradation of particulate primary production.

In subsystem II, a more refractory pool appears to also support bacterial growth. PhOx and EEAleu were the only enzymes that could explain any variability in BSP in II (Table 4). Positive relations of these enzymes with BSP here imply that organic matter degraded by PhOx and EEAleu could have become relatively more important as a source and promoted bacterial growth and abundance in subsystem II. Other studies in different environments support our findings. Lignin material led to increased bacterial abundance in soils where the bacterial population assimilated lignin-derived carbon (Deangelis et al., 2011). Also in peatlands bacterial growth was linked to phenol oxidase activity (Fenner et al., 2005). In freshwater, PhOx activity is less explored (Münster and De Haan, 1998) but bacteria seem to play a leading role in decomposing lignin in aquatic ecosystems (Li et al., 2008). Lignin degradation is a relatively slow process (Benner and Kaiser, 2011) and significantly increases with higher temperature (Donnelly et al., 1990). Because lake water typically has a long residence time, such waters can be important places for microbial processing of carbon received from terrestrial sources. In river systems, occasional flood events are especially important in this process; they deliver significant amounts of low-age, terrestrial DOM (Berggren et al., 2009). We propose that semi-isolated floodplain side arms (such as subsystem II) provide opportunities for microbial processing of more refractory carbon. Sporadic inputs of fresh terrestrial material, reduced hydrodynamic stress due to rare flooding, high oxygen availability and the stimulating effect of phytoplankton on PhOx synthesis may create favorable conditions that could trigger the utilization of lignin-derived material.

Despite the elevated % PER in subsystem III, no direct link between BSP and pPP or PER was observed. This could be because bacteria in that subsystem did not take up algae-derived carbon directly. Our results, however, imply that BSP was driven by primary production here. This was found also in lakes with terrestrial input, where bacterial metabolism was driven by autochthonous primary production (Kritzberg et al., 2005). Even since mean pelagic primary production was significantly lower in III compared to the highly productive floodplain side arms of subsystem I, PER was still able to sustain bacterial metabolism also in subsystem III. In our study, close and positive relations of EEAb and BSP (Table 4) suggest that PER products, hydrolyzed by EEAb (Figure 7E), supported BSP. The activity of β–glucosidase is strongly stimulated by carbohydrates, which phytoplankton accumulates as a storage material (Mallet and Debroas, 2001; Børsheim et al., 2005). This may imply that β-linked polysaccharides derived from phytoplankton degradation could be beneficial sources sustaining bacterial growth in subsystem III. This allows us to suggest that in isolated floodplain lakes, despite high terrestrial input, bacterial growth can be also driven by autochthonous carbon sources to a certain extent (Robertson et al., 1999).

### Extracellular enzymatic activity linking primary and secondary production

Our results stress the importance of primary production in sustaining BSP in backwaters with a connectivity gradient. Nonetheless, the only modest variation of BSP compared to more dynamic primary production (pPP, PER, and chl *a*) demonstrates that organic matter derived from sources other than phytoplankton also supported BSP in this river-floodplain system. The potential coupling between bacterial and phytoplankton components can be indicated by the BSP:PPt ratio (Van Wambeke et al., 2002). This ratio is a measure for the importance of heterotrophic bacteria in consuming material from primary production (Kirchman, 2010). A high ratio could reflect significant input of terrestrial OM (Kirchman, 2012). The highest value in subsystem III implies that allochthonous, non-planktonic sources were relatively more important than bacterial utilization of phytoplankton-derived C (compare Tranvik, 1989; Gao et al., 2007). EEA measurements, however, point to phytoplankton exudates as an important source supporting BSP in subsystem III. Our study demonstrates that, despite the lack of data for a direct relationship between bacterial and phytoplankton components, the EEA analyses are able to elucidate the patterns relating these two variables, especially in systems with high terrestrial input.

Bacteria can utilize an increasing proportion of primary production, when phytoplankton is less efficient (Conan et al., 1999). At increased primary productivity, when phytoplankton is most active, the BSP:PPt ratio is the lowest (Almeida et al., 2005; Morana et al., 2014). The lowest ratio has been sometimes interpreted as a low flux of labile OM to bacteria (Ducklow et al., 2012), hence indicating a weak coupling between the bacterial and phytoplankton component. In our study, however, the lowest BSP:PPt ratio in subsystem I was accompanied by a strong coupling of BSP and pPP. Moreover, EEA measurements emphasize the significance of primary production (pPP, PER, and chl *a*) for bacterial utilization. Hence, we suggest that in subsystem I a fast transfer of phytoplankton-derived carbon to bacteria occurred: at elevated primary production, more of phytoplankton-derived material was assimilated by bacteria (compare De Kluijver et al., 2014). Our conclusion is that EEA measurements are very useful in describing the coupling of bacterial and phytoplankton components. The BSP:PPt ratio alone does not fully describe the strength of this link (Morán et al., 2002). Drawing conclusions based solely on that ratio simplifies the mechanisms that relate these two components (Almeida et al., 2005) potentially overlooking important links between bacteria and phytoplankton.

### Conflict of interest statement

The authors declare that the research was conducted in the absence of any commercial or financial relationships that could be construed as a potential conflict of interest.
