# Supplementary figures and images for: Algal extracellular release in river-floodplain dissolved organic matter: response of extracellular enzymatic activity during a post-flood period
Source: Front Microbiol. 2015 Feb 17;6:80. doi: 10.3389/fmicb.2015.00080 (PMC4330910; doi:10.3389/fmicb.2015.00080)

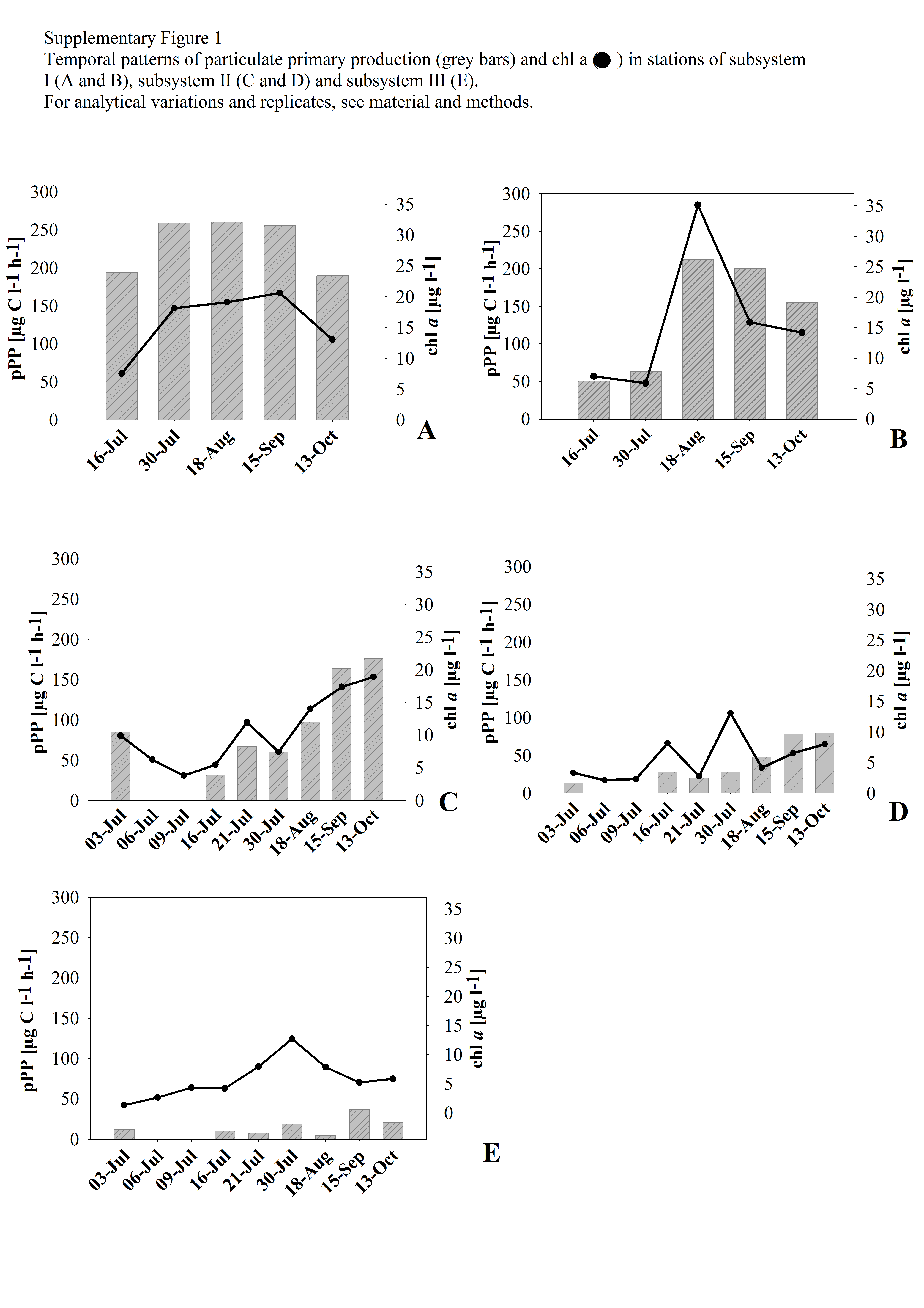

Supplement: Supplementary file 1 [file Image1.TIF]

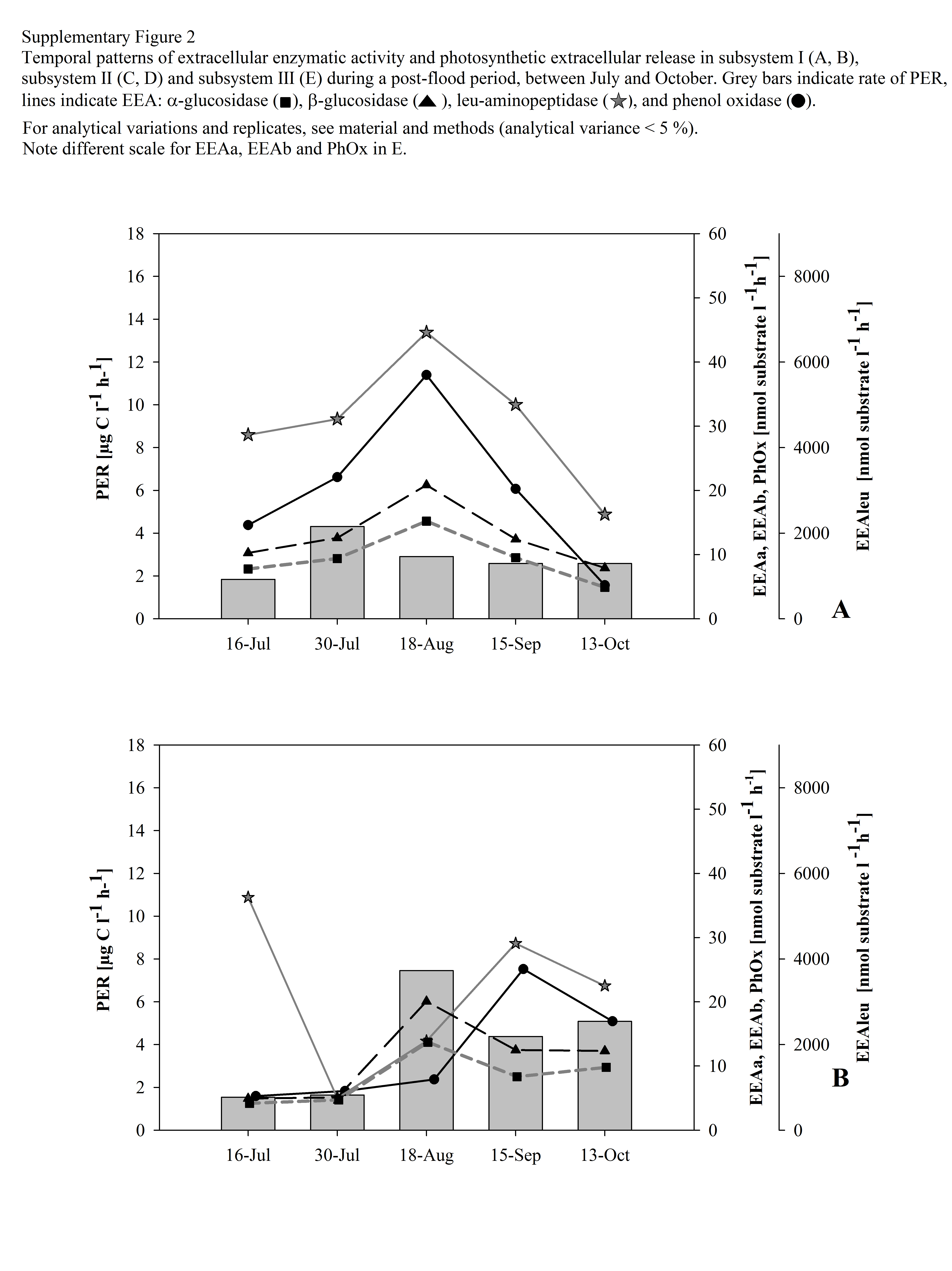

Supplement: Supplementary file 2 [file Image2.TIFF]

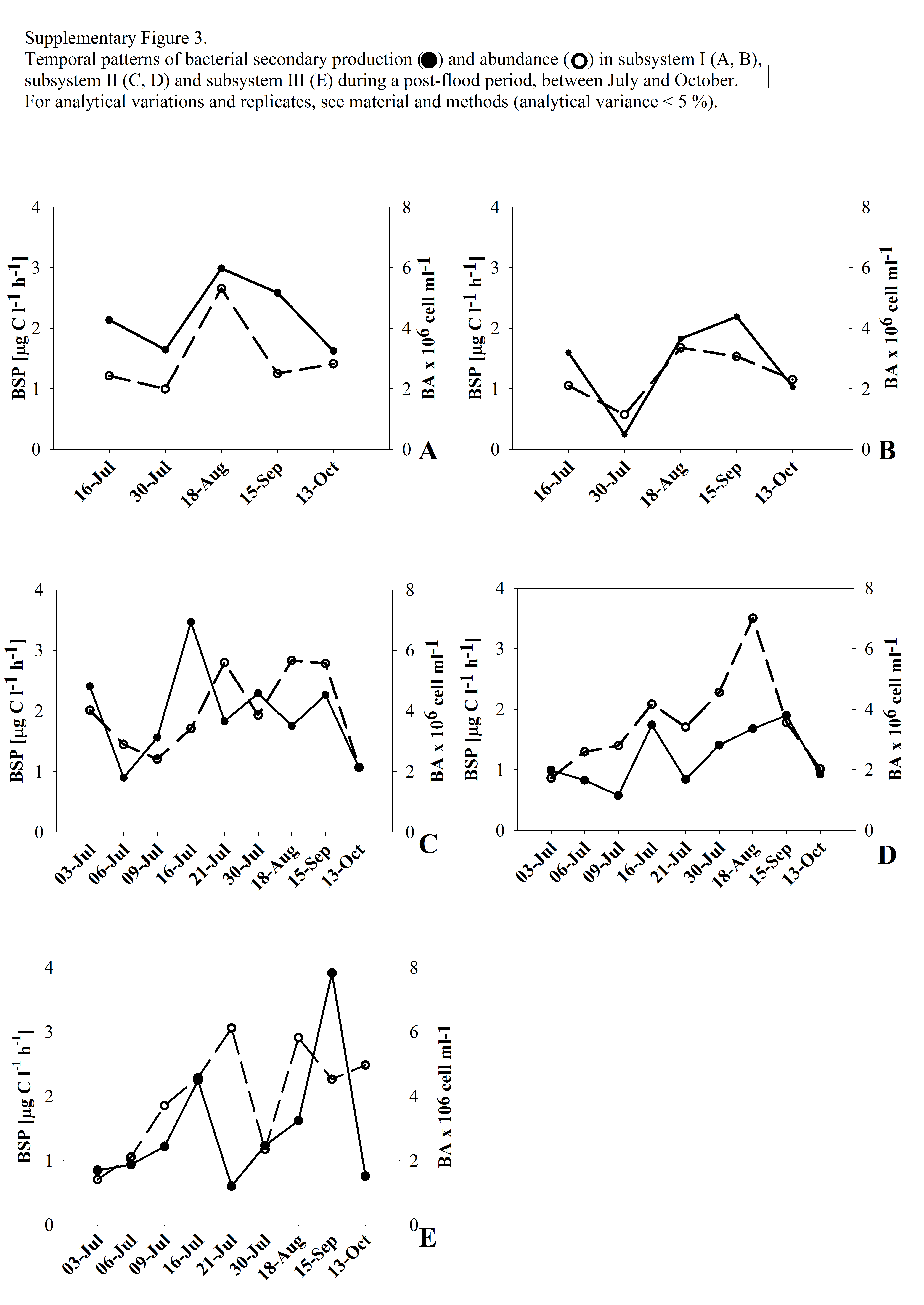

Supplement: Supplementary file 3 [file Image3.TIF]
